# Supplementary material for: Spatial Heterogeneity and Temporal Trends in Malaria on the Thai–Myanmar Border (2012–2017): A Retrospective Observational Study
Source: Trop Med Infect Dis. 2019 Apr 12;4(2):62. doi: 10.3390/tropicalmed4020062 (PMC6630951; doi:10.3390/tropicalmed4020062)
Supplement: Supplementary file 1 [file tropicalmed-04-00062-s001.zip › Supplementary Figure S1 Tabel S1.pdf]

Raw rates: Thai

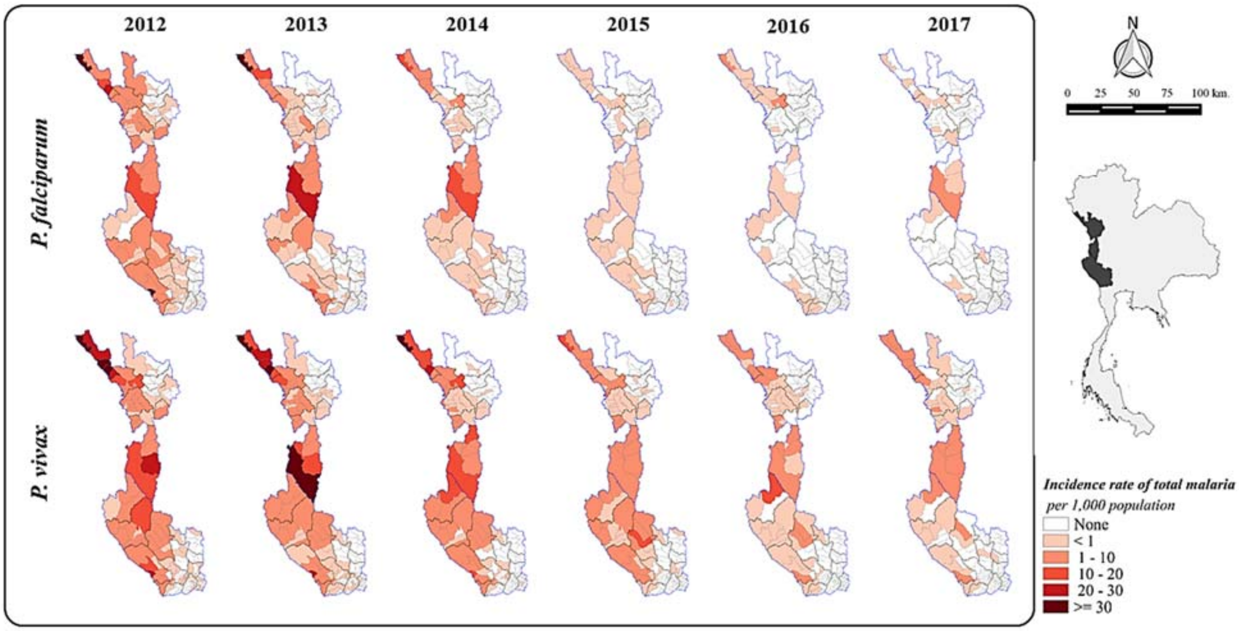

Figure S1 Raw rates map of *P. falciparum* and *P. vivax* among Thai citizens

Table S1 Number of sub-district in each interval of *P. falciparum* and *P. vivax* incidence rates per 1,000 population per year by <u>raw rates</u>

Incidence rates	2012	2013	2014	2015	2016	2017
P. falciparum						
**0**	77	94	115	125	137	145
**< 1**	41	42	32	36	22	15
**1 - 10**	38	21	12	0	2	1
**10 - 20**	2	2	2	0	0	0
**20 - 30**	1	1	0	0	0	0
**≥ 30**	2	1	0	0	0	0
P. vivax						
**0**	66	72	92	90	102	111
**< 1**	46	43	27	37	40	31
**1 - 10**	35	35	32	32	18	19
**10 - 20**	6	5	8	2	1	0
**20 - 30**	5	3	1	0	0	0
**≥ 30**	3	3	1	0	0	0
