# Supplementary material for: Spatial Heterogeneity and Temporal Trends in Malaria on the Thai–Myanmar Border (2012–2017): A Retrospective Observational Study
Source: Trop Med Infect Dis. 2019 Apr 12;4(2):62. doi: 10.3390/tropicalmed4020062 (PMC6630951; doi:10.3390/tropicalmed4020062)
Supplement: Supplementary file 1 [file tropicalmed-04-00062-s001.zip › Supplementary Figure S3.pdf]

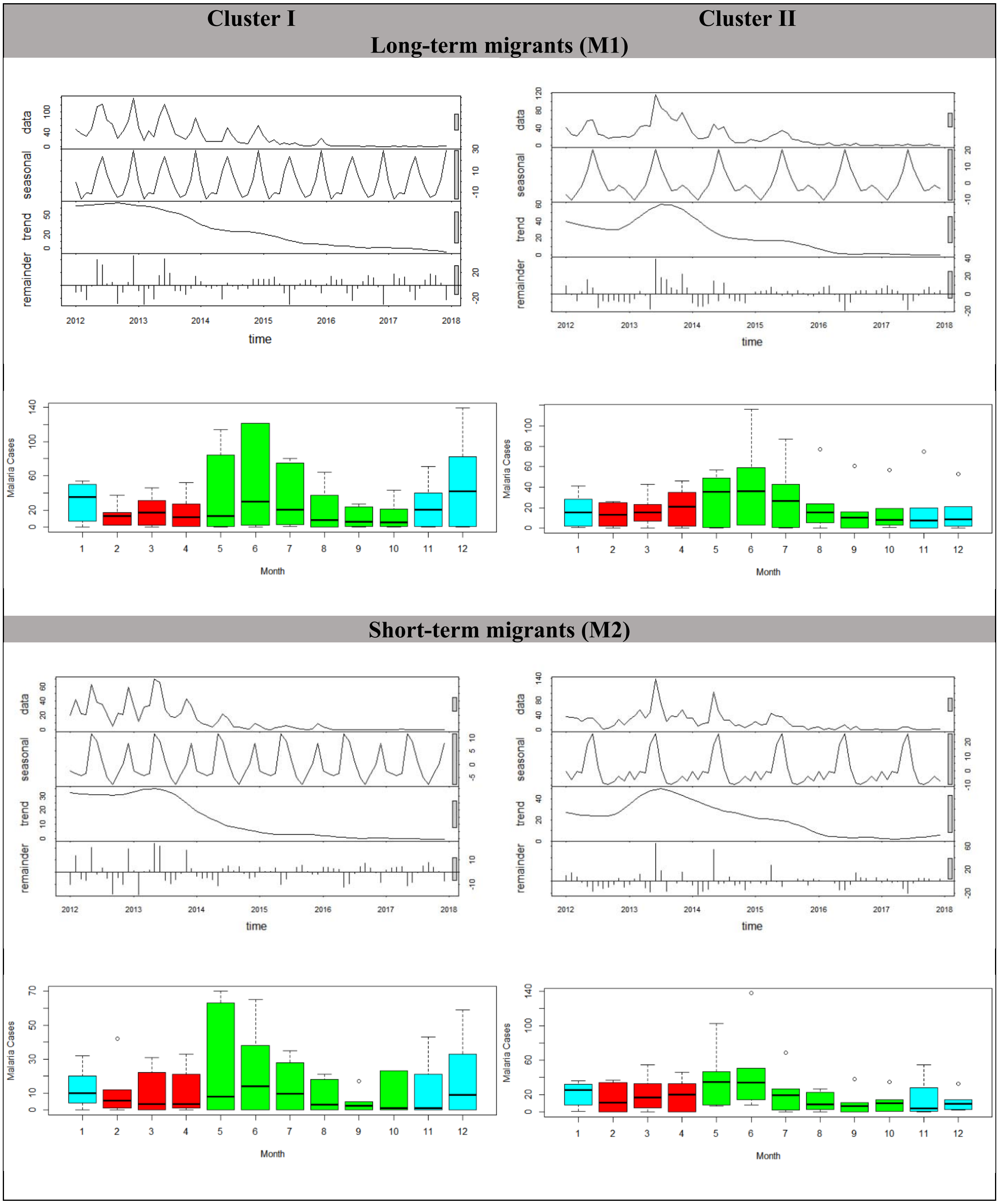

Figure S2 STL and seasonal patterns of *P. falciparum* cases among M1 and M2 between 2012 and 2017 in Cluster I and Cluster II. Color of box plot; blue represents the cold season, red represents hot season, and green represents the rainy season.
