# Supplementary figures and images for: Spatial Heterogeneity and Temporal Trends in Malaria on the Thai–Myanmar Border (2012–2017): A Retrospective Observational Study
Source: Trop Med Infect Dis. 2019 Apr 12;4(2):62. doi: 10.3390/tropicalmed4020062 (PMC6630951; doi:10.3390/tropicalmed4020062)

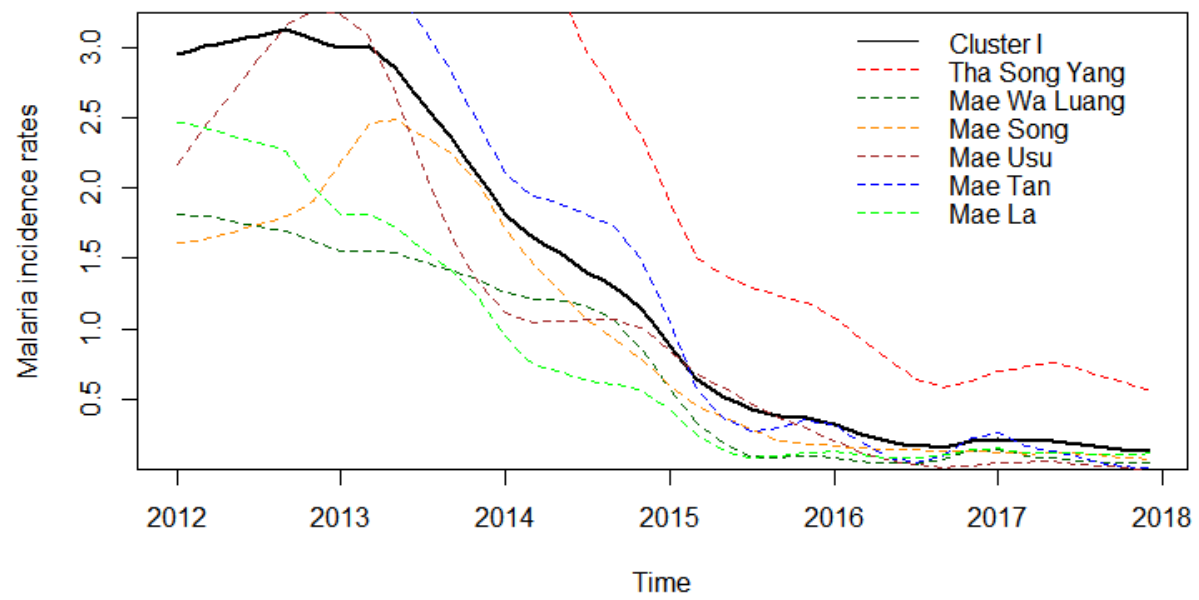

**Figure S4** Trend of *P. vivax* cases among Thai citizens between 2012 and 2017 in Cluster I (referred to **Figure 5 (c1)**)

Supplement: Supplementary file 1 [file tropicalmed-04-00062-s001.zip › Supplementary Figure S5.pdf]

*P. falciparum*

M1

M2

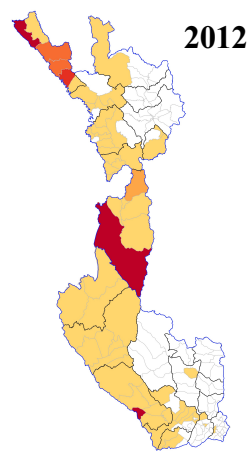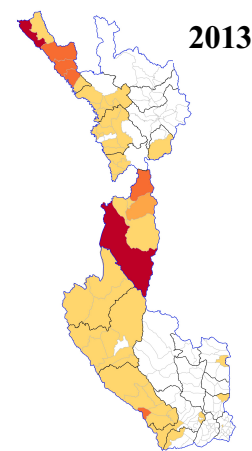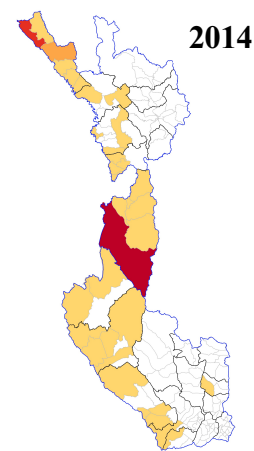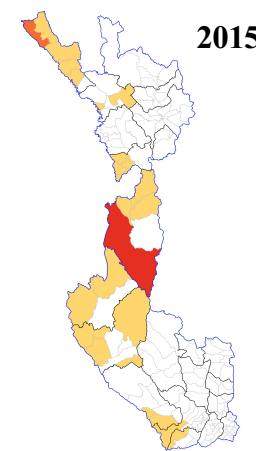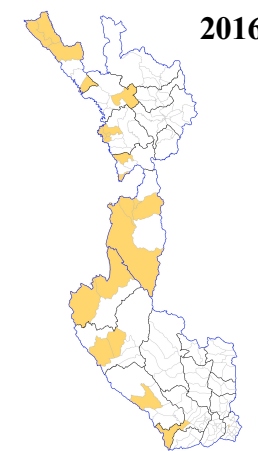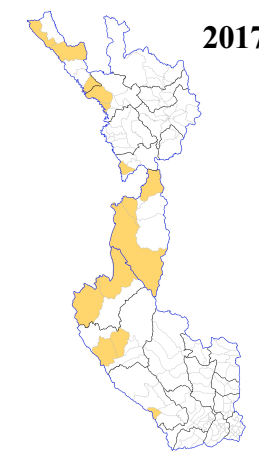

a

Number of cases

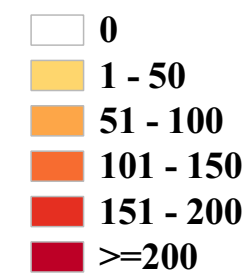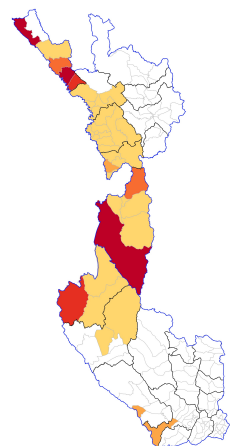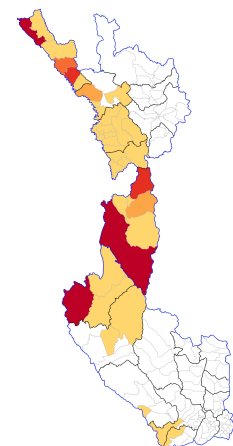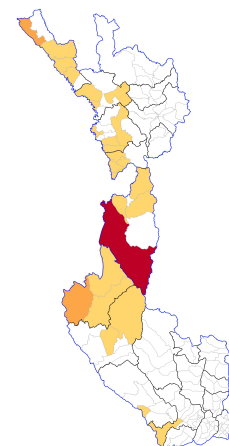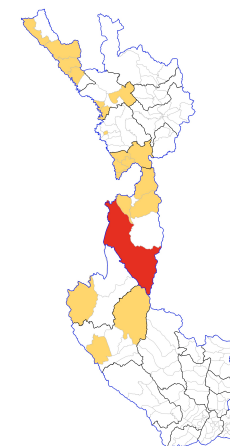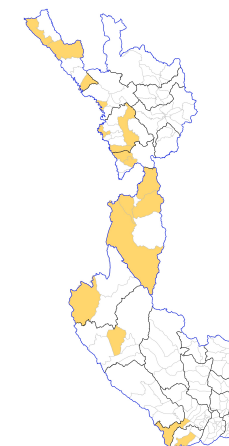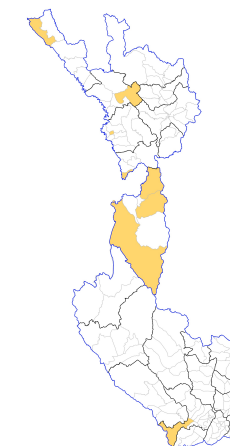

*P. vivax*

M1

M2

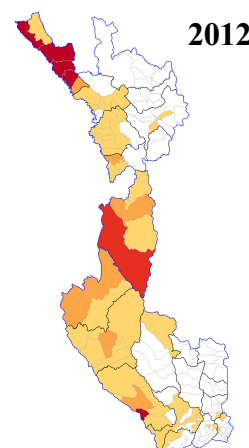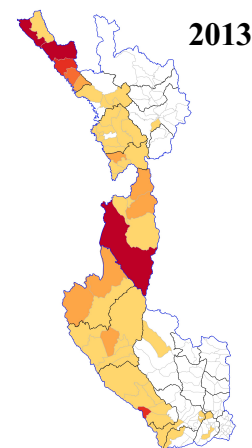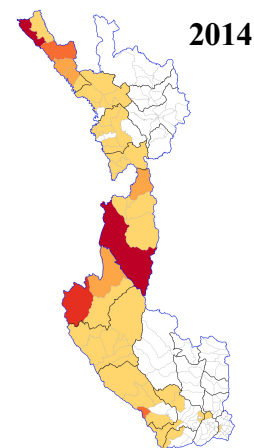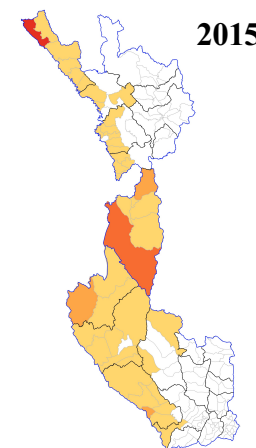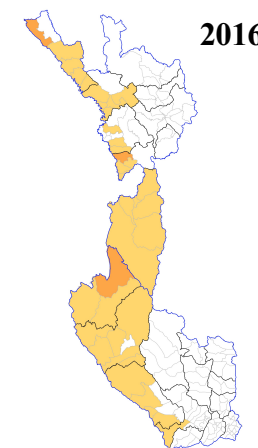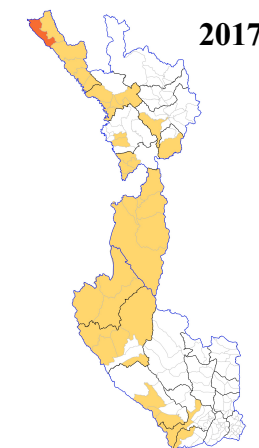

b

Number of cases

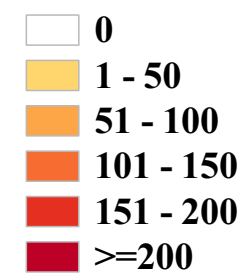

Supplement: Supplementary file 1 [file tropicalmed-04-00062-s001.zip › Supplementary Figure S2.pdf]
